# Psychedelic drug use and schizotypy in young adults

**DOI:** 10.1038/s41598-021-94421-z

**Published:** 2021-07-23

**Authors:** Alexander V. Lebedev, K. Acar, B. Garzón, R. Almeida, J. Råback, A. Åberg, S. Martinsson, A. Olsson, A. Louzolo, P. Pärnamets, M. Lövden, L. Atlas, Martin Ingvar, P. Petrovic

**Affiliations:** 1 Department of Clinical Neuroscience, Karolinska Institutet, K8 Klinisk neurovetenskap, K8 Neuro Ingvar, 171 77 Stockholm, Sweden; 2 Aging Research Center, Karolinska Institutet and Stockholm University, Stockholm, Sweden; 3 Stockholm University Brain Imaging Center (SUBIC), Stockholm University, Stockholm, Sweden; 4 Department of Psychology, New York University, New York, USA; 5 Department of Psychology, University of Gothenburg, Göteborg, Sweden; 6 National Center for Complementary and Integrative Health, National Institutes of Health, Bethesda, MD USA; 7 National Institute on Drug Abuse, National Institutes of Health, Baltimore, MD USA; 8 National Institute of Mental Health, National Institutes of Health, Bethesda, MD USA

**Keywords:** Computational neuroscience, Epidemiology, Psychosis

## Abstract

Despite recently resurrected scientific interest in classical psychedelics, few studies have focused on potential harms associated with abuse of these substances. In particular, the link between psychedelic use and psychotic symptoms has been debated while no conclusive evidence has been presented. Here, we studied an adult population (n = 1032) with a special focus on young (18–35 years) and healthy individuals (n = 701) to evaluate the association of psychedelic drug use with schizotypy and evidence integration impairment typically observed in psychosis-spectrum disorders. Experimental behavioural testing was performed in a subsample of the subjects (n = 39). We observed higher schizotypy scores in psychedelic users in the total sample. However, the effect size was notably small and only marginally significant when considering young and healthy subjects (Cohen’s d = 0.13). Controlling for concomitant drug use, none of our analyses found significant associations between psychedelic use and schizotypal traits. Results from experimental testing showed that total exposure to psychedelics (frequency and temporal proximity of use) was associated with better evidence integration (Cohen’s d = 0.13) and a higher sensitivity of fear responses (Cohen’s d = 1.05) to the effects instructed knowledge in a reversal aversive learning task modelled computationally with skin conductance response and pupillometry. This effect was present even when controlling for demographics and concomitant drug use. On a group level, however, only difference in sensitivity of fear responses to instructed knowledge reached statistical significance. Taken together, our findings suggest that psychedelic drug use is only weakly associated with psychosis-like symptoms, which, in turn, is to a large extent explained by psychiatric comorbidities and use of other psychoactive substances. Our results also suggest that psychedelics may have an effect on flexibility of evidence integration and aversive learning processes, that may be linked to recently suggested therapeutic effects of psychedelic drugs in non-psychotic psychiatric populations.

## Introduction

Classical serotonergic psychedelics refer to a set of psychoactive substances that produce profound effects on mood and high-order cognition primarily via agonism on the serotonin system^1^ (1). Examples of such psychedelics are LSD (lysergic acid diethylamide), psilocybin, DMT (*N*,*N*-dimethyltryptamine) and mescaline. After a 40-year hiatus in biomedical research, scientists have recently started to re-visit their therapeutic value. In the modern era of “psychedelic renaissance”, we are witnessing an intensification of basic neuroscientific and clinical research of these drugs, showing promising, although preliminary, results for various psychiatric conditions^2–4^, as well as for overall wellbeing and life satisfaction in healthy people^5^. In contrast, research of their potentially harmful effects remains limited. In addition to this renewed scientific and therapeutic interest in psychedelic substances, the recreational use of these substances has increased substantially and is almost as common today as it was in the 1960s^6^.

Although psychedelics are considered to be physiologically safe, negative psychological effects are possible and may be considerable^1,7^. One particularly debated issue is the link between psychedelic use and psychosis-spectrum disorders. Specifically, for a long time there has been a worry that psychedelics may trigger development of prolonged psychotic reactions^8^. In light of this, it is worth mentioning that despite the fact that the first studies (n = 2588) did find a link between psychedelic use and psychosis-like symptoms^9^, larger studies (n > 130,000) failed to confirm this^10^, neither could the use of these drugs be associated with other mental health problems^11^. One of the possible explanations for the discrepancies between these studies may be that different strategies were used to assess psychopathology. Indeed, diagnosis-oriented assessments typically employed in larger population studies may not be ideal to detect subclinical manifestations of psychopathological features, including non-clinical schizotypal traits, cognitive biases typical for schizophrenia-spectrum, such as Bias Against Disconfirmatory Evidence (BADE)^12,13^ and aberrations in learning^14–16^.

The present study aimed to bridge this knowledge gap by investigating whether past psychedelic use in a healthy non-clinical young population is associated with abnormalities in higher-order cognition, similar to the ones displayed by schizophrenia-spectrum individuals (specifically, Bias Against Disconfirmatory Evidence and impairments in reversal aversive learning). Attempting to address some of the limitations of cross-sectional designs and to tease-out potential causal links, an extended assessment of drug use patterns was also carried out in a follow-up survey, collecting information about total exposure to different drugs, by asking question about frequency and recency of use. Specifically, we submitted the hypothesis that psychedelic drug use is associated with schizotypy symptoms and cognitive biases typical for schizophrenia-spectrum disorders and that total exposure to these drugs has significant effects on the afore-mentioned outcomes.

## Methods

### Sample and overall design

The study was approved by the Swedish Ethical Board (Regionala Etikprövningsnämnden i Stockholm, DNR: 2018/1040-31) and adhered to the principles of the Declaration of Helsinki. An electronic informed consent was obtained from all the participants prior to screening and all of the tested participants additionally provided written informed consent before the experimental data collection.

The study incorporated a cross-sectional design, and studied a population of Swedish adults with a special focus on healthy young adults. The required sample size was determined with G*Power v3 for small and medium-to-large effect sizes for the main sample and the experimental arm, respectively.

Recruitment occurred through web-based announcements on social media services, forums which were expected to include our target population (Facebook and Reddit groups discussing scientific and recreational use of psychedelic drugs, drug policy, substance-related issues and disorders), as well as general platforms designed to recruit research subjects. Data were collected through a survey (n = 1032), and behavioural testing (BADE and fear reversal learning) conducted in a subset of subjects (n = 39). Table 1 shows a list of assessments in the survey. See Supplement S1 for a flow chart of the overall study design.

**Table 1:** Study samples.

Sample	Screening	Follow-up	Testing
N (total/healthy young adults)	701/951	197/260	39
Assessments	O-LIFE, PDI, EPI, history of drug use	Drug use (extended)	RALT, BADE
Education^a^, years: median(range)	3 (0–35)	3 (0–12)	4 (0–12)
Age, years: mean ± SD	26.94 ± 4.59	26.3 ± 4.65	27.21 ± 4.83
Sex: %F	69.3	63.96	46.15
Ever used: alcohol	92.74	90.86	84.62
Ever used: tobacco	70.77	70.56	76.92
Ever used: cannabis	55.31	62.94	69.23
Ever used: psychedelics	28.29	36.55	56.41
Ever used: MDMA	31.34	38.58	38.46
Ever used: stimulants	30.49	33.5	28.21
Ever used: opiates^b^	12.51	13.2	10.26
O-LIFE total: median (range)	15 (0–37)	14 (0–36)	15 (4–35)
PDI total: median (range)	6 (0–22)	6 (0–19)	6 (0–17)

Sample overview, descriptive statistics characterize population that provided necessary demographic data and met study criteria.

*O-LIFE* 43-item Oxford-Liverpool Inventory of Feelings and Experiences, *PDI+* extended 21-item Peters Delusion Inventory, which also includes 5 additional questions (items 24, 33, 34, 41 and 42) pertaining to unusual experiences from CAPE-42 (Community Assessment of Psychic Experience), *EPI* – Ego Pathology Inventory (not used in the present study), *RALT* – reversal aversive learning task, *BADE* Bias Against Disconfirmatory Evidence.

^a^Post high school; ^b^including non-recreational use.

The survey employed screening for a number of psychopathology-spectrum traits (see complete list in the Open Science Foundation [OSF] entry: https://osf.io/2wbcx). The present study focused specifically on schizotypy in relation to psychedelic drug use. The subjects were explicitly asked not to associate the symptoms with the acute effects of psychoactive drugs when filling out the questionnaires. A follow-up survey was sent to all participants who specified their e-mail address in the initial survey and assessed extended history of drug use.

As we were specifically interested in the relation between psychedelics and psychosis-related symptoms in young adults that have no psychiatric or neurological comorbidities, a set of additional criteria was applied to delineate this group:

- Age between 18 and 35 years
- No neurological, psychiatric (such as major depression, bipolar disorder, schizophrenia, attention deficit hyperactivity disorder, autism-spectrum disorder, obsessive–compulsive disorder) or serious medical illness
- No history of head trauma or brain damage

An experimental arm of the study was formed from a randomly drawn subset of psychedelic users without history of psychiatric disorders who live in Stockholm area (n = 22) and their age- and sex-matched non-user counterparts (n = 17) meeting the afore-mentioned criteria.

Schizotypy was assessed with 43-item Oxford-Liverpool Inventory of Feelings and Experiences, O-LIFE^17,18^ and a 21-item Peters Delusion Inventory PDI^19^, which also included five additional questions (items 24, 33, 34, 41 and 42) related to unusual experiences from a 42-item CAPE^20^. The results were also confirmed with a composite score merging effects of OLIFE and a standard, 21-item version of PDI. Adult ADHD Self-Report Scale^21^ and The Ritvo Autism Asperger Diagnostic Scale^22^ were also used to assess symptoms of attention deficit hyperactivity disorder and autism, respectively. As a part of another project, The Scharfetter's Ego Pathology Inventory^23^ was administered to evaluate prevalence depersonalisation-derealization phenomena in the studied population.

## Primary outcome

### Survey

The primary study outcome collected in the survey was a questionnaire-based measure of schizotypy calculated as a composite z-score that merges effects of PDI + and O-LIFE (see OSF entry: https://osf.io/2wbcx).

### Experimental tasks

#### Bias against disconfirmatory evidence

In the BADE task^13^, the subjects are asked to rate the plausibility of four different interpretations of a scenario, three times. Twelve of the scenarios are emotional, twelve neutral, and the remaining six are distractors. The emotional-neutral scenarios are presented with three sequentially disambiguating hints, and plausibility ratings are gathered after each hint. Two of the four interpretations are Lures (initially plausible but require revision as new hints are given), one is Absurd (highly implausible and remains so throughout the scenario), and one is True (initially moderately plausible but gradually becomes the most plausible interpretation). See Supplement S2 for a detailed illustration of a scenario.

The component taken into account during scoring of the task was *Evidence* *Integration* *Impairment* (EII), which characterizes lack of ability to modify beliefs when facing new information. EII was calculated from ratings of plausibility for different scenarios as recommended by Sanford et al.^24^:

$$ Absurd\;1 + Absurd\;2 + Absurd\;3 + Lure - A\;3 + Lure - B\;3 - True\;3 $$

In plain text, EII was computed as the sum of the plausibility ratings for Absurd interpretations gathered after each one of the three hints, plus the sum of plausibility ratings of Lure-A and Lure-B interpretations gathered after the last hint, minus the plausibility rating of the True interpretation gathered after the last hint.

#### Fear reversal learning task

**Figure 1:**
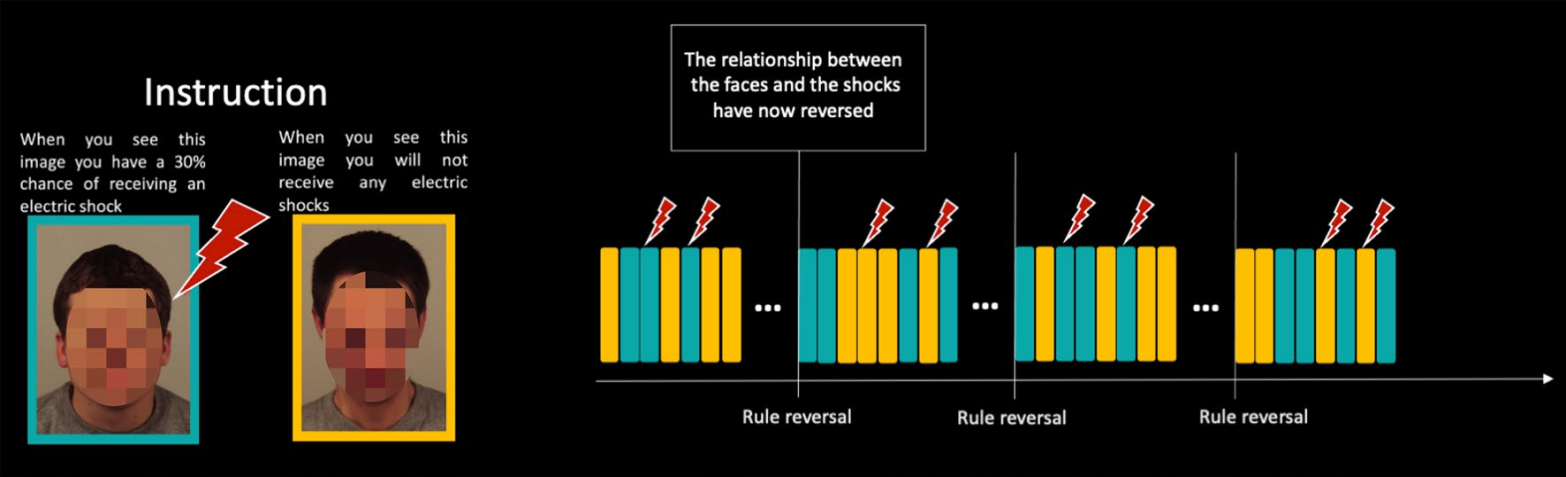
Instructions and block design of the aversive fear learning task.

Subjects underwent an instructed aversive fear reversal learning task as described by Atlas et al.^25^, in which mild electric shocks were used as unconditioned stimulus (US) and conditioned stimuli (CS) were two angry faces from the Karolinska Directed Emotional Faces database^26^. Prior to the task, US intensity was individually determined by gradually increasing shock-intensity to a level where subjects deemed the shocks to be highly unpleasant, but still tolerable. The task consisted of four blocks, and each block consisted of four trials of the CS coupled with the US, eight trials of the CS without electric shocks (CS+), and eight trials of the stimulus that were not coupled with electric shocks (CS−) (i.e. 30% reinforcement rate). After rule reversal, CS+ became CS−, and vice versa. Rule reversals occurred three times during the task (every 20 trials). See Fig. 1 for illustration of the task. All subjects were explicitly informed about contingencies and rule reversals before each block with a short text (example in Fig. 1). Trials were pseudorandomized with two requirements; two shocks were never delivered twice in a row, and that the same condition never occurred three times in a row. The subjects’ fear response during CS presentation was measured by means of pupillometry and skin conductance response (SCR). The peak change was measured in an eight-second window following CS offset relative to a value measured 500 ms before the stimulus (Supplement S4). The task lasted for about 16 min.

Detailed description of the experimental setting and computational modelling approach is available in the Supplement S11.

### Analysis

Representativeness of the subsamples was evaluated employing analysis of variance with sampling as a grouping factor. All the subsamples (including the experimental arm) were representative of the main screened sample in terms of severity of schizotypy, ADHD and autism-spectrum symptoms. Descriptive data is presented in Table 1.

Primary outcome was checked for normality with the Shapiro–Wilk test and screened for outliers based on 1.5*interquartile range (IQR). In cases of slight deviations from normality, assessment of the residuals was additionally carried out prior to conducting parametric tests. The analysis leveraged general linear modelling as implemented in R Programming Environment, version 3.6^27^ adjusting for demographics, psychiatric diagnoses, concomitant drug use. Direct group comparisons of the mean scores were also carried-out post-hoc.

### Reproducibility statement

All data analysis steps and models are documented in R-scripts available at the study GitHub repository https://github.com/kasimacar/HUD. The *rstan* code for the implemented modified Rescorla–Wagner model is available at the https://github.com/alex-lebedev/ILDPII repository as “RW_instr_multipleSubj_initFree.stan” (“/stan_models” folder).

The study was preregistered at the Open Science Foundation database (OSF entry: https://osf.io/2wbcx, 10.17605/OSF.IO/2WBCX).

## Results

### Survey: psychedelic use and schizotypy

A total of 1032 individuals were screened for the initial survey (total sample) and 701 of them were between 18 and 35 years old (*M* ± *SD* = 27.99 ± 6.26), had no neurological, psychiatric or serious medical illness nor any history of head trauma or brain damage (young healthy sample).

No outliers were detected and most of the outcome variables passed the distribution normality criteria. Data from a final sample of 1032 subjects were used in the analysis.

In a multiple linear regression model adjusting for concomitant drug use, the association between schizotypy and psychedelic use was not significant. This was true both for the whole sample (n = 1032, *β*(*SE*) = 0.003(0.02), *t*(*1021*) = 0.191, *p* = 0.85) and for the population of young adults without neurological and psychiatric disorders (n = 701, *β*(*SE*) = 0.0004(0.02), *t*(*691*) = 0.024, *p* = 0.98). In line with this, we did not find any significant links between total exposure to psychedelics (frequency and temporal proximity) and elevated schizotypy scores in a subsample of subjects who completed an extended evaluation of their drugs use patterns (n = 197, see Supplement S7). In contrast, stimulant use (cocaine, amphetamines, ephedrine) strongly and consistently predicted higher scores of schizotypy (*β*(*SE*) = 0.057(0.02), *t*(*691*) = 2.8, *p* = 0.005), whereas alcohol use was associated with lower scores (*β*(*SE*) =  − 0.04(0.01), *t*(*691*) = − 2.78, *p* = 0.005). *Pseudo-R*^*2*^ (*Cragg–Uhler*) *of* *the* *model* *was* 0.09, AIC = 530.15. This also held true in the sample of all screened subjects (*Pseudo-R*^*2*^ = 0.09, AIC = 777.39) for stimulant use (*β*(*SE*) = 0.065(0.02), *t*(*1021*) = 3.87, *p* = 0.0001), but not for alcohol use (*β*(*SE*) =  − 0.02(0.01), *t*(*1021*) =  − 1.76, *p* = 0.08).

In a model including psychiatric diagnosis and psychedelic use as regressors in the total sample, only diagnosis had significant association with schizotypy (n = 1032, *β*(*SE*) = 0.075(0.01), t(1028) = 6.82, *p* < 0.001), whereas diagnosis-by-group (users vs non-users) interaction effect on schizotypy was non-significant (n = 1032, *β*(*SE*) = 0.007(0.01), t(1028) = 0.69, *p* = 0.49).

**Figure 2:**
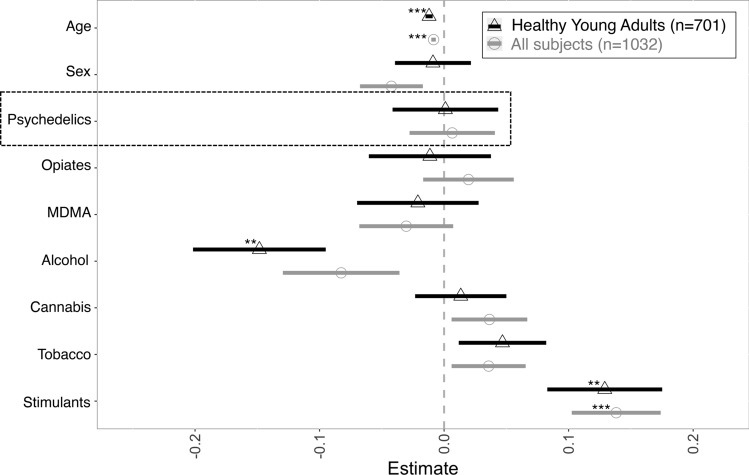
Drug use and schizotypy. Regression coefficient plot of models with all screened subjects included (n = 1032), and only subjects that met study criteria (n = 701). **p < 0.01, ***p < 0.001.

Addressing sampling bias in the regression models found no significant impact of sampling sites on any of the reported results. The findings therefore did not support the presence of a substantial link between psychedelic use and subclinical manifestations of the investigated psychopathological features in otherwise healthy subjects. See Fig. 2 for regression coefficient plots of both models. Tables for both models can be found in Supplement S7.

Addressing the multidimensionality of schizotypy^28^ we also conducted similar multiple regression analyses focusing on four major facets of the construct (Supplement S8a–c): unusual experiences (UE), cognitive disorganisation (CD), introversive anhedonia (IA) and impulsive nonconformity (IN). The yielded associations were in line with the main result showing no significant associations between psychedelic use and schizotypy when adjusted for concomitant drug use, psychiatric diagnosis and demographics. Similarly, the only drug class that was consistently associated with higher schizotypy scores was stimulants.

Despite the lack of significant associations between psychedelic use and schizotypy in multiple regression models, it is worth noting that direct group comparisons revealed that psychedelic users (*n* = 323) on average scored significantly higher on schizotypy compared to non-users (*n* = 709) with a small effect size (Cohen’s *d* [95% CI] = 0.16 [0.025 0.29], *t*_*one-tailed*_ (649) = 2.38, *p* = 0.009), see Supplement S6. Similarly, the results showed marginally significant difference in schizotypy between the groups with a small effect-size, when specifically considering young healthy subjects (Cohen’s *d* [95% CI] = 0.13 [0.035 0.30], *t*_*one-tailed*_ (319) = 1.58, *p* = 0.06).

### Experimental arm

In line with the confirmed representativeness of the experimental arm, psychedelic users generally scored higher on most of the facets of schizotypy, but likely due to power limitations the difference reached statistical significance only for the facet “impulsive nonconformity” (*t*_*one-tailed*_ (33.2) = 2.25, p_uncorr_ = 0.03).

### Bias against disconfirmatory evidence

Apart from direct group comparison, a multiple linear regression analysis was conducted, controlling for concomitant drug use and demographics, in order to test whether exposure (composite z-score merging effects of frequency and proximity of drug use) to psychedelics is associated with more marked evidence integration impairment (EII) in the BADE task. Thirty-seven out of the 39 subjects (22 psychedelic drug users and 17 sex/age-matched non-users) were included in the analyses; one subject did not complete the task, and a second subject was excluded due to computer failure during ongoing task.

**Figure 3:**
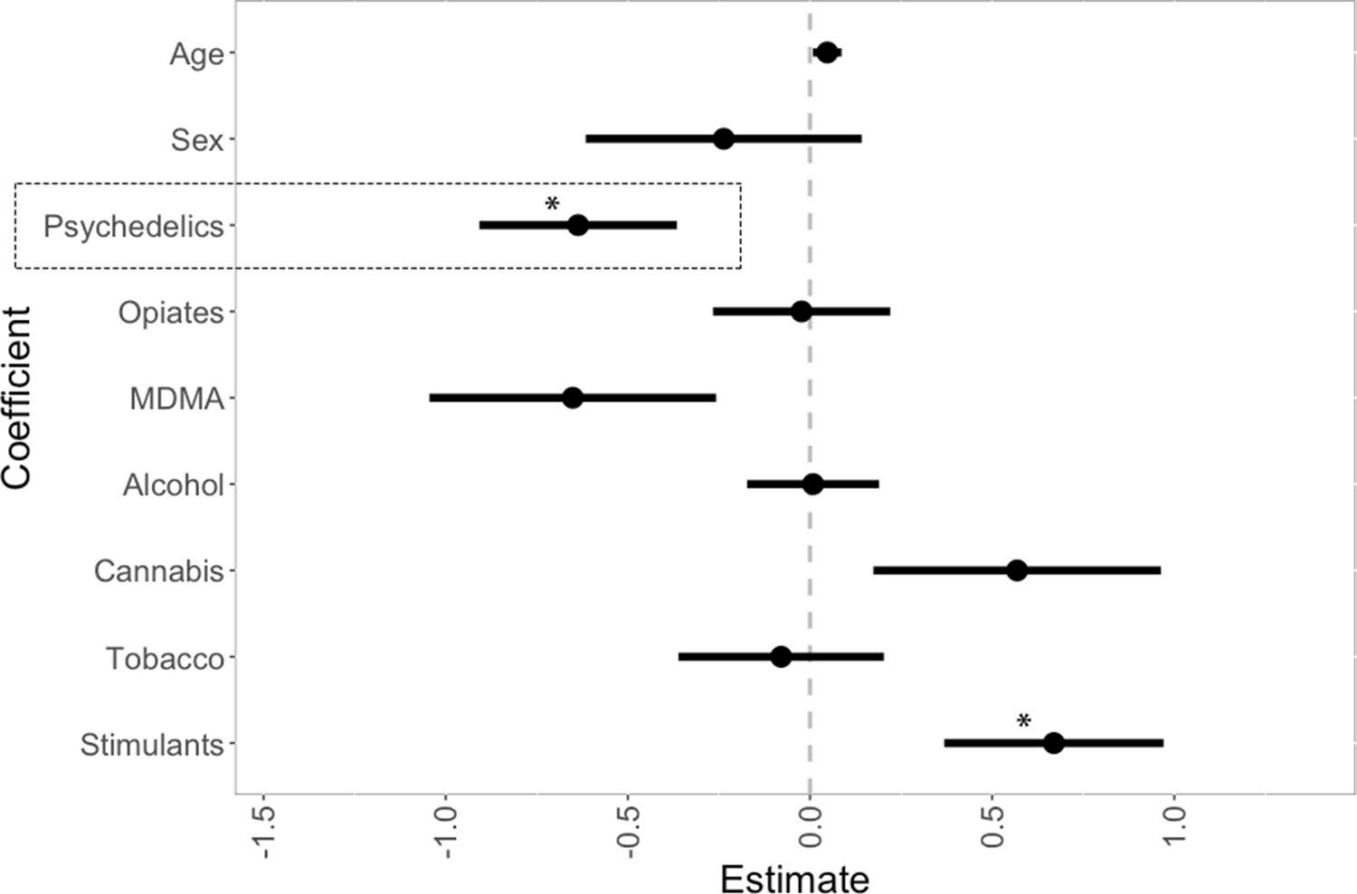
Drug use and evidence integration impairment (EII). Regression coefficient plot of exposure to drugs and demographics as predictors of evidence integration impairment. *p < 0.05.

On a group level, psychedelic users scored lower on EII compared to their non-user counterparts, but this difference in means did not reach statistical significance, m_users_(SD) = − 0.19 (± 1.04), m_non-users_(SD) = 0.26 (± 0.91), p = 0.17. Multiple regression analysis, however, showed that psychedelic exposure significantly predicted lower scores of EII (*β* = − 120.41, *t*(27) = − 2.35, *p* = 0.03, Cohen’s *d* [95% CI] = 0.13 [− 0.33, 0.6]), while stimulant exposure significantly predicted higher scores (*β* = 113.11, *t*(27) = 2.22, *p* = 0.04). MDMA exposure did not predict lower scores, but showed such tendencies (*β* = − 113.12, t(27) =  − 1.66, *p* = 0.11, Cohen’s *d* [95% CI] = 0.06 [− 0.53, 0.4]). The overall model fit was *R*^2^ = 0.31, AIC = 504. See Fig. 3 for coefficient plot of the model. Table can be found in Supplement S3.

### Aversive fear learning task

**Figure 4:**
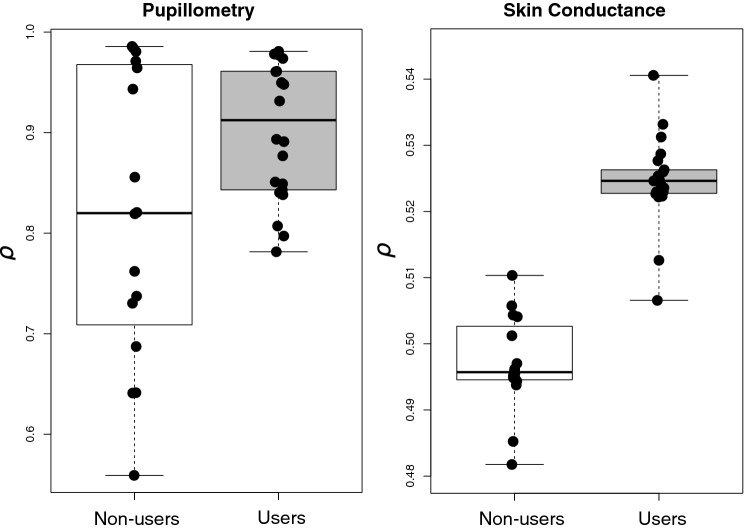
Effects of instructed rule reversals on fear learning (*ρ*) in subjects with and without history of psychedelic drug use.

One subject was excluded from the analyses after a request to discontinue the task. One more subject did not exhibit skin conductance response and therefore was excluded from the corresponding analyses. Thus, a total of 38 subjects who had usable pupillometry data (n_non-users_ = 16, n_users_ = 22) and 37 with SCR data (n_non-users_ = 16, n_users_ = 21) to estimate the model parameters. Overall, pupillometry-derived estimates of expected value dynamics fitted the data better (R-squared_marginal/conditional_ = 0.21/0.22) than the ones derived with SCR (R-squared_marginal/conditional_ = 0.005/0.22), which, in turn, may explain generally lower estimates of the *ρ*–parameter (Fig. 4) in the latter model. See Supplement S9 and S10 for more detailed description of the model fit and estimated parameters.

Two-sample t-tests showed that psychedelic users were more influenced by instructions about rule reversals. This was true for the ρ-parameter estimated with pupillometry data: Cohen’s *d* [95% CI] = 0.82 [0.11, 1.52], *t* = 2.20, *p* = 0.04, and this between-group difference was even larger for the parameter estimated with the skin conductance response: Cohen’s *d* [95% CI] = 4.1 [2.5, 5.68], *t* = 11.9, *p* < 0.001. See Fig. 4. Additionally, a linear regression analysis showed that overall exposure to psychedelics (temporal proximity and frequency of use) was associated with larger effects of instructed knowledge, on pupillometry-derived ρ-parameter (*β*(*SE*) = 0.04(0.02), *t*(36) = 2.37, *p* = 0.02, Cohen’s *d* [95% CI] = 1.05 [0.56, 1.54]).

In multiple regression analyses controlling for demographics and concomitant drug use, overall exposure to psychedelics consistently exhibited positive association with higher sensitivity of fear responses to instructed knowledge (Supplement S5).

## Discussion

The present study found that psychedelic users scored significantly higher on schizotypy compared to controls. However, the effect-size was notably low and when excluding all participants with history of psychiatric diagnoses, this difference was no longer significant (albeit a threshold significance remained). This is in line with a large population study, which failed to demonstrate the abovementioned association^10^, but contradicts an older study by Kuzenko et al., which did demonstrate an association between use of these substances and psychotic symptoms^9^. However, it is worth noting that in the study by Kuzenko et al. the identified significant effects of psychedelic use were not adjusted for stimulant use despite the fact that the latter was also strongly associated with psychotic symptoms, in line with our findings. Our study did not find evidences for detrimental effects of these differences on wellbeing. On the contrary, psychedelic users scored lower on the ‘disturbance’ facet of the Peters Delusion Inventory. With our results, however, we cannot completely rule out a possibility of potentially detrimental effects of psychedelic use on other psychiatric and wellbeing dimensions. Future meta-analyses employing assessment of moderators and potential biases should shed more light on this matter.

Contrary to the submitted hypothesis, exposure to psychedelics was associated with better evidence integration in the BADE task, indicating a greater readiness of psychedelic users to re-adjust initial plausibility ratings when faced with disconfirmatory evidence. These findings support the rationale of psychedelic-assisted therapy for non-psychotic psychiatric conditions characterized by overly fixed cognitive styles, such as, for example, depression^2,4,29^.

In contrast, we found that stimulant exposure was significantly associated with worse evidence integration mirroring its association with schizotypy identified in the present and previous studies^30^, as well as in a non-clinical sample of subjects that scored high on delusion-proneness^12^.

Psychedelic users also exhibited higher sensitivity to instructed knowledge in the fear learning task. Notably, these effects of instructions were positively associated with overall level of exposure to psychedelics, i.e., more recent and frequent intake of psychedelics was associated with even greater influence of instructions on fear responses. This suggests that psychedelics may augment top-down fear learning in a lasting way, which, in turn, may explain their particular efficacy in treating anxiety and trauma-related psychiatric disorders^31,32^. An alternative explanation, however, could still be that there is a third factor associated both with likelihood of psychedelic drug use and higher flexibility of fear learning in these subjects. One candidate for this can be a personality trait openness, which has previously been shown to be generally higher among psychedelic drug users^33^ and be influenced by psychedelic experiences in experimental settings^34,35^. This is also of a particular relevance for the identified between-group differences in sensitivity to the instructed reversals, as interactions between openness and trust has been reported in previous studies^36^.

The findings from the testing arm of the study are novel in that we have for the first time behaviourally demonstrated that higher-order evidence integration and fear learning flexibility are associated with history of psychedelic use and exposure to these drugs. However, these findings remain to be further investigated in experimental studies together with the underlying neural mechanisms.

Several limitations need to be considered when interpreting the results. Despite made efforts to address causal relationships by evaluating overall exposure to drugs with variables of interest it is important to acknowledge the retrospective nature of these ratings. Although all subjects with history of psychiatric disorders were excluded from the experimental parts of the study, no information was collected on psychiatric disorders of closest relatives. Interpretation of the survey results might also be limited by the self-report nature of the collected measures and recall biases. Addressing sampling bias in the regression models, however, found no significant impact of sampling sites on the results. Another important subject for discussion is the assessment of schizotypy, which is currently recognized as a multidimensional construct^28,37^. In the present study, however, we used a composite score merging effects of different facets of this construct, which was done primarily due to the inconsistent literature on the association between specific domains of schizotypy and psychedelic drug use in order to maximize the statistical power by avoiding the need to correct for multiple tests of different facets. Meanwhile, we address this limitation in the follow-up analyses of the subdomains of OLIFE showing consistent results with no relationships with psychedelic use and strongest associations observed between stimulant use and unusual experiences. Finally, it is worth noting that the present analysis was fully focused on schizopyty and did not take into account other traits such as symptoms of autism-spectrum and attention deficit hyperactivity disorders, which often co-occur with schizotypy-related traits^38^. The future analyses focusing on broader spectra of symptoms will address these associations.

To conclude, our analyses did not support the hypothesis that psychedelics may pose serious risks for developing psychotic symptoms in healthy young adults. On the contrary, the use and overall exposure to these drugs was associated with better evidence integration, and more flexible aversive learning. Future experimental studies might provide further clarification of causal relationships between the investigated traits and effects of these substances. It is also important to note that the lack of a strong relation between use of psychedelics and psychosis-associated symptoms does not preclude that such drugs are detrimental for individuals with a high risk of developing psychotic disorders—an important question that needs to be investigated in future studies.

## Supplementary Information

Supplementary Information 1.

## Data Availability

All data used in the main analyses are available as supplementary materials (‘HUD_data_anonymized.xlsx’). Raw pupillometry and SCR data, as well as the derived trial-by-trial responses can be requested from the authors and transferred for specific analysis projects that are in line with the original ethical approval. This requires a data use agreement, which effectively transfers the confidentiality obligations of the institution (Karolinska Institutet) at which the original research was conducted to the institution of the recipient of the data.
