# Supplementary material for: Psychedelic drug use and schizotypy in young adults
Source: Sci Rep. 2021 Jul 23;11:15058. doi: 10.1038/s41598-021-94421-z (PMC8302700; doi:10.1038/s41598-021-94421-z)
Supplement: Supplementary file 1 — Supplementary Information 1. [file 41598_2021_94421_MOESM1_ESM.docx]

**Supplement**

**Psychedelic drug use and schizotypy in young adults.**

Lebedev AV*^1^, Acar K*^1^, Garzón B^2,5^, Almeida R^3^, Råback J^1^, Åberg A^1^, Martinsson S^1^, Olsson A^1^, Louzolo A^1^, Pärnamets P^1,4^, Lövden M^2,5^, Atlas L^6,7,8^, Martin Ingvar^1^, Petrovic P^1^

* - equal contribution

^1^ Department of Clinical Neuroscience, Karolinska Institutet, Sweden

^2^ Aging Research Center, Karolinska Institutet & Stockholm University, Sweden

^3^ Stockholm University Brain Imaging Center (SUBIC), Stockholm University, Sweden

^4^ Department of Psychology, New York University, USA

^5^ Department of Psychology, University of Gothenburg, Sweden

^6^ National Center for Complementary and Integrative Health, National Institutes of Health, Bethesda, MD

^7^ National Institute on Drug Abuse, National Institutes of Health, Baltimore, MD, USA

^8^ National Institute of Mental Health, National Institutes of Health, Bethesda, MD, USA

## **Supplement S1.** Study Flow Chart.


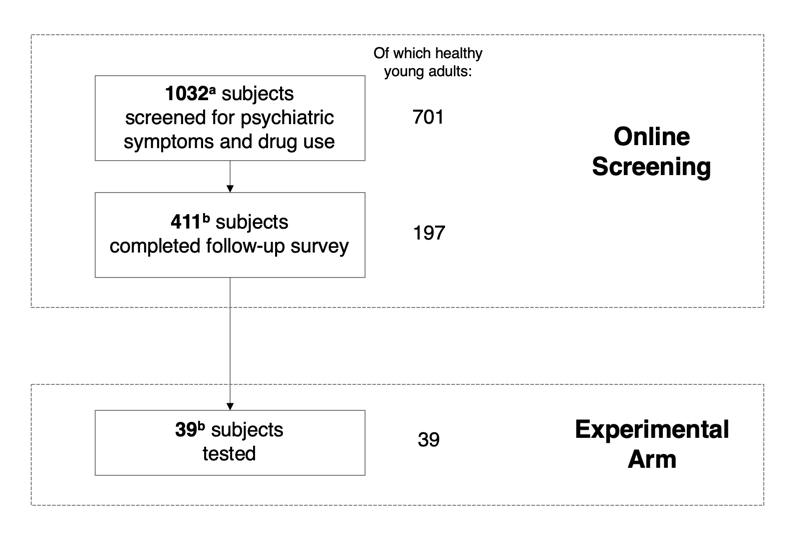


^a^ **Primary outcomes collected:** O-LIFE – 43-item Oxford-Liverpool Inventory of Feelings and Experiences; PDI – 26-item Peters Delusion Inventory;

^b^ **Secondary outcomes collected:** RALT – Reversal Aversive Learning Task; BADE – Bias Against Disconfirmatory Evidence.

**Supplement S2.** Example of the BADE task

An example of a scenario: “Jenny can’t fall asleep”, with the interpretations: “Jenny is excited about Christmas morning” (True),“Jenny is nervous about her exam the next day” (Lure-A), “Jenny is worried about her ill mother” (Lure-B), and “Jenny loves her bed” (Absurd). The true interpretation initially does not seem to be very plausible, but becomes more so after each hint. Plausibility ratings are gathered on a scale from 0-10 with a scroll bar underneath each interpretation. Six distracter-scenarios are included in order to prevent subjects from employing a pattern of responding. In the distracter scenarios, the True interpretation is apparent after the first hint. (See Figure S2 below)


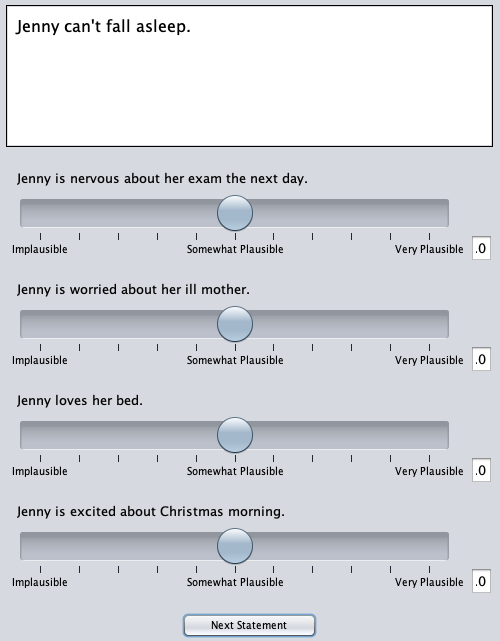

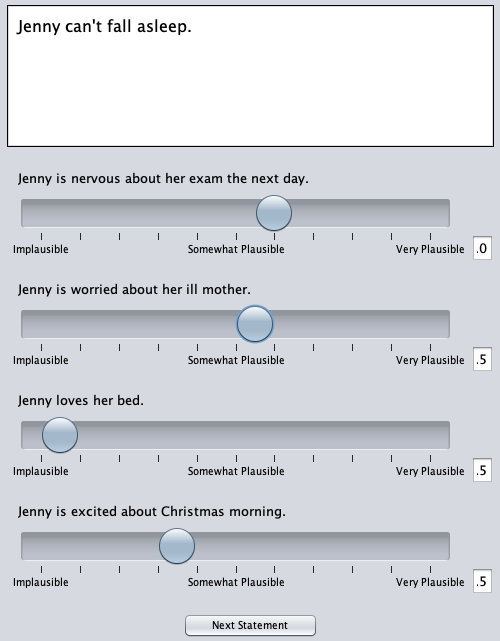


Start of a scenario with the first hint First interpretation of the first hint


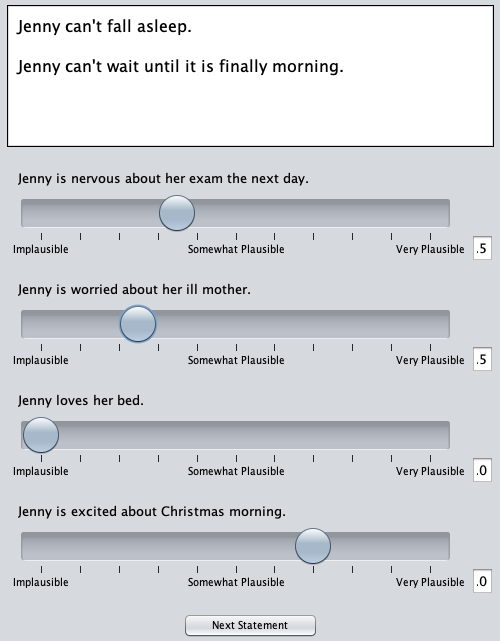

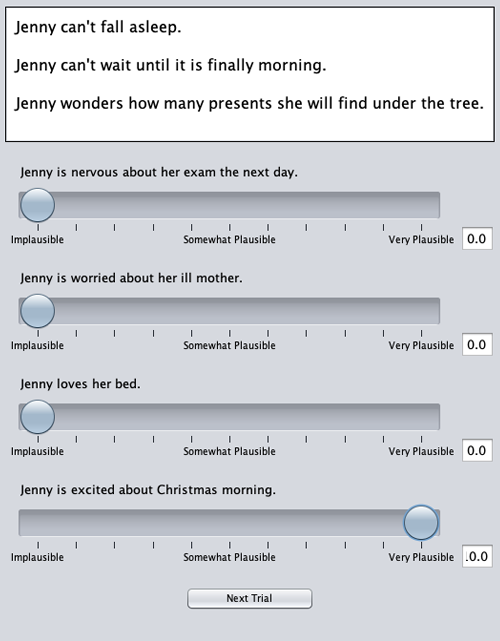


Second hint, and second interpretation Last hint, and last interpretation

**Supplement S3.** Psychedelic Use and Bias Against Disconfirmatory Evidence: Tables

| *Regression results using EII as the criterion* | | | | | | |
| --- | --- | --- | --- | --- | --- | --- |
| *Predictor* | *β* | *b* | *b*  *95% CI*  *[LL, UL]* | *sr^2^* | *sr^2^*  *95% CI*  *[LL, UL]* | *p* |
| (Intercept) |  | -65.86 | [-515, 383] |  |  | 0.765 |
| Age | 44.33 | 9.43 | [-6.66, 25.52] | .04 | [-.06, .14] | 0.239 |
| Sex (man) | -23.78 | -47.06 | [-201, 107] | .01 | [-.04, .06] | 0.537 |
| Psychedelics | -120.41 | -126.53 | [-237, -15.95] | .14 | [-.05, .33] | 0.027* |
| Opiates | -3.29 | -4.58 | [-103, 94.67] | .00 | [-.01, .01] | 0.925 |
| MDMA | -113.12 | -129.45 | [-289, 31.09] | .07 | [-.07, .21] | 0.110 |
| Alcohol | 1.99 | 1.67 | [-72.29, 75.64] | .00 | [-.00, .00] | 0.963 |
| Cannabis | 105.75 | 113.01 | [-47.94, 273.95] | .05 | [-.07, .17] | 0.161 |
| Tobacco | -15.23 | -15.72 | [-130, 99.34] | .00 | [-.02, .03] | 0.781 |
| Stimulants | 113.11 | 133.07 | [10.22, 255] | .13 | [-.05, .31] | 0.035* |
| Note: A significant *b*-weight indicates the semi-partial correlation is also significant. *β* represents standardized regression weights. *sr^2^* represents the semi-partial correlation squared. *LL* and *UL* indicate the lower and upper limits of a confidence interval, respectively.  * *p* < .05. | | | | | | |

**Supplement S4.** Illustration of the sampling window of pupil diameter.

**
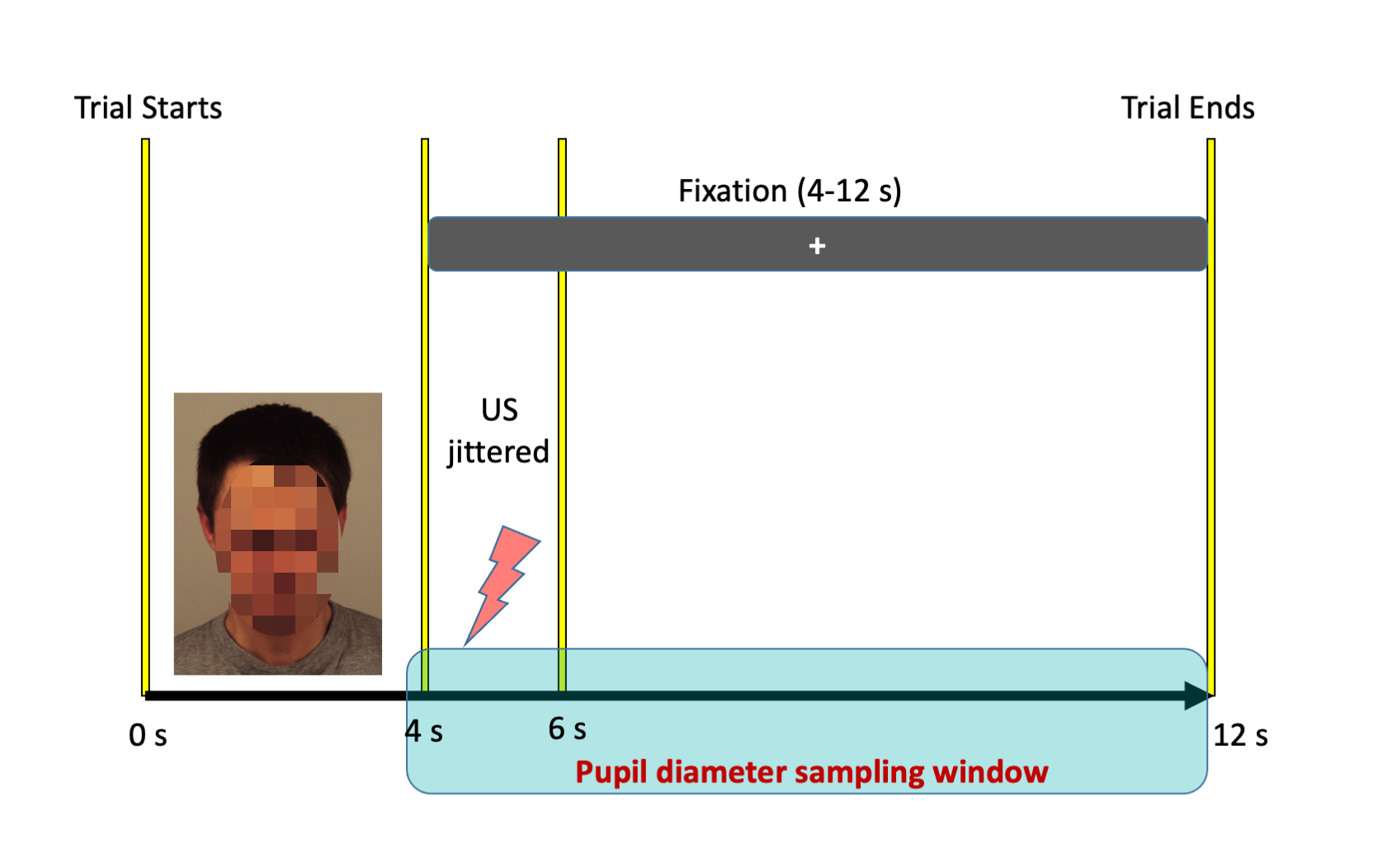
**

**Supplement S5.** Effects of drugs exposure on flexibility of fear responses (pupillometry and SCR-derived rho-parameter).


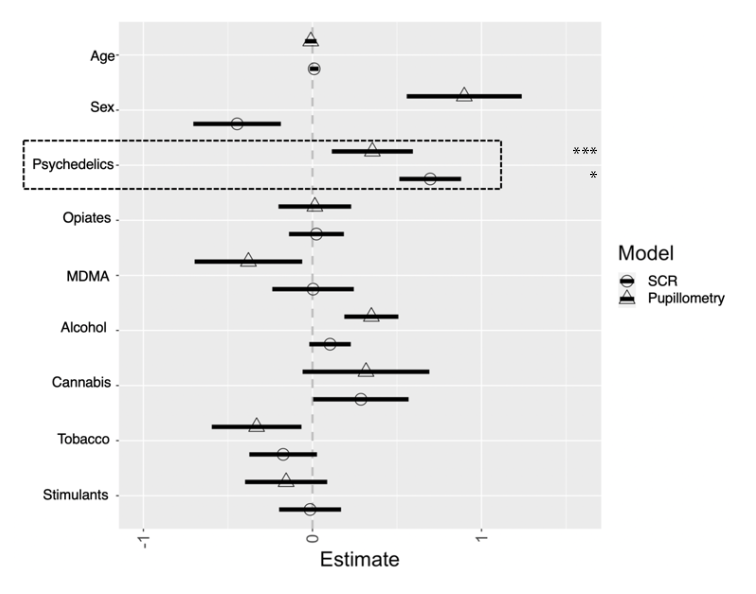


**Supplement S6.** Psychedelic Use and Schizotypy: Group Comparison

*
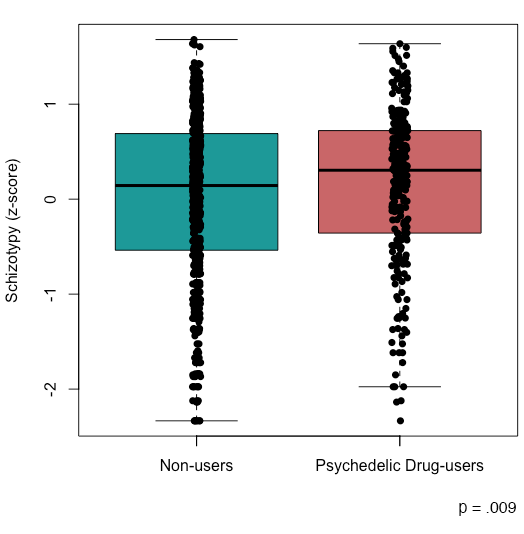
*

Box and whiskers plot of between-group differences in schizotypy scores. The box represents the IQR; The line in the middle of the box is the median. Top and bottom of the box represents upper (Q3) and lower (Q1) quartiles, respectively. Upper and lower whiskers represent Q3 + 1.5 x IQR and Q1 – 1.5 x IQR, respectively.

**Supplement S7.** Psychedelic Use and Schizotypy: Regression Tables

**
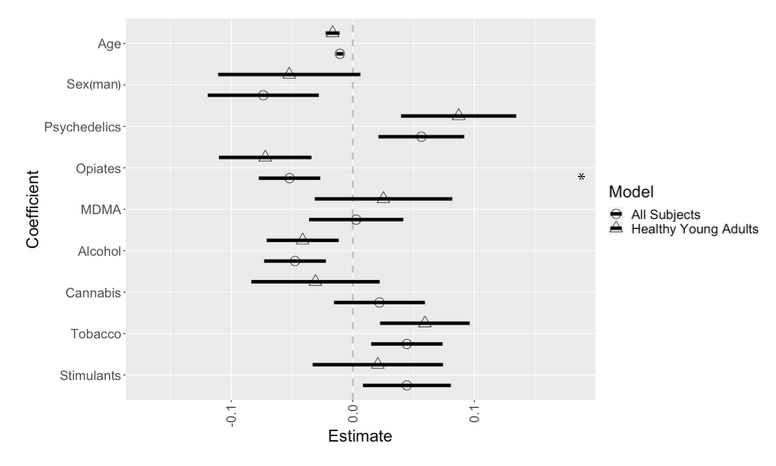
**Regression coefficient plot of models with Schizotypy as the criterion, and total overall drug exposure as predictors. With all screened subjects included (n=360), and only subjects that met study criteria (n=197).

| Regression results using Schizotypy as an outcome and total overall drug exposure as predictors. Only subjects with no reported history of psychiatric diagnoses included (N = 197). | | | | | | | |
| --- | --- | --- | --- | --- | --- | --- | --- |
| *Predictor* | *b* | | *β* | *b*  *95% CI*  *[LL, UL]* | *sr^2^* | *sr^2^*  *95% CI*  *[LL, UL]* | *p* |
| (Intercept) | 1.38 |  | | [1.07, 1.69] |  |  |  |
| Age | -0.02 | | -0.08 | [-0.03, -0.01] | .04 | [-.01, .09] | 0.004*** |
| Sex (man) | -0.05 | | -0.03 | [-0.17, 0.06] | .00 | [-.01, .02] | 0.37** |
| Psychedelics | 0.09 | | 0.08 | [-0.01, 0.18] | .02 | [-.02, .05] | 0.07 |
| Opiates | -0.07 | | -0.05 | [-0.15, 0.00] | .02 | [-.02, .05] | 0.06 |
| MDMA | 0.03 | | 0.02 | [-0.09, 0.14] | .00 | [-.01, .01] | 0.66 |
| Alcohol | -0.04 | | -0.04 | [-0.10, 0.02] | .01 | [-.02, .03] | 0.17 |
| Cannabis | -0.03 | | -0.03 | [-0.13, 0.07] | .00 | [-.01, .01] | 0.56 |
| Tobacco | 0.06 | | 0.06 | [-0.01, 0.13] | .01 | [-.02, .04] | 0.11 |
| Stimulants | 0.02 | | 0.02 | [-0.09, 0.13] | .00 | [-.01, .01] | 0.70 |
| Note: A significant *b*-weight indicates the semi-partial correlation is also significant. *β* represents standardized regression weights. *sr^2^* represents the semi-partial correlation squared. *LL* and *UL* indicate the lower and upper limits of a confidence interval, respectively. ** *p* < .01, *** *p* < .001 | | | | | | | |

| *Regression results using Schizotypy as the criterion, all subjects included (N = 1032).* | | | | | | |
| --- | --- | --- | --- | --- | --- | --- |
| *Predictor* | *b* | *β* | *b*  *95% CI*  *[LL, UL]* | *sr^2^* | *sr^2^*  *95% CI*  *[LL, UL]* | *p* |
| (Intercept) | 0.75 |  | [0.37, 1.14] |  |  |  |
| Age | -0.02 | -0.11 | [-0.04, -0.01] | .02 | [.00, .03] | 0.000*** |
| Sex (man) | -0.08 | -0.04 | [-0.21, 0.05] | .00 | [-.00, .01] | 0.23 |
| Psychedelics | 0.05 | 0.02 | [-0.12, 0.22] | .00 | [-.00, .00] | 0.55 |
| Opiates | 0.07 | -0.02 | [-0.12, 0.26] | .00 | [-.00, .00] | 0.47 |
| MDMA | -0.15 | -0.07 | [-0.34, 0.04] | .00 | [-.00, .01] | 0.13 |
| Alcohol | -0.25 | -0.06 | [-0.48, -0.01] | .02 | [-.00, .01] | 0.04* |
| Cannabis | 0.08 | -0.04 | [-0.07, 0.23] | .00 | [-.00, .01] | 0.32 |
| Tobacco | 0.09 | -0.04 | [-0.06, 0.23] | .00 | [-.00, .01] | 0.24 |
| Stimulants | 0.37 | 0.17 | [0.19, 0.54] | .02 | [.00, .03] | 0.000*** |
| Note: A significant *b*-weight indicates the semi-partial correlation is also significant. *β* represents standardized regression weights. *sr^2^* represents the semi-partial correlation squared. *LL* and *UL* indicate the lower and upper limits of a confidence interval, respectively. * *p* < .05, *** *p* < .001 | | | | | | |

| *Regression results using Schizotypy as the criterion. Only subjects with no reported history of psychiatric diagnoses included (N = 701).* | | | | | | | |
| --- | --- | --- | --- | --- | --- | --- | --- |
| *Predictor* | *b* | | *β* | *b*  *95% CI*  *[LL, UL]* | *sr^2^* | *sr^2^*  *95% CI*  *[LL, UL]* | *p* |
| (Intercept) | 0.95 |  | | [0.51, 1.39] |  |  |  |
| Age | -0.03 | | -0.14 | [-0.04, -0.02] | .02 | [.00, .05] | 0.00*** |
| Sex (man) | -0.03 | | -0.01 | [-0.18, 0.12] | .00 | [-.00, .00] | 0.68 |
| Psychedelics | 0.04 | | 0.02 | [-0.16, 0.24] | .00 | [-.00, .00] | 0.71 |
| Opiates | 0.01 | | 0.00 | [-0.23, 0.25] | .00 | [-.00, .00] | 0.95 |
| MDMA | 0.10 | | -0.04 | [-0.33, 0.14] | .00 | [-.00, .01] | 0.42 |
| Alcohol | -0.35 | | -0.10 | [-0.61, -0.09] | .01 | [-.00, .02] | 0.008** |
| Cannabis | -0.00 | | -0.00 | [-0.18, 0.17] | .00 | [-.00, .00] | 0.98 |
| Tobacco | -0.09 | | 0.04 | [-0.08, 0.25] | .00 | [-.00, .01] | 0.32 |
| Stimulants | 0.35 | | 0.16 | [0.13, 0.57] | .01 | [-.00, .03] | 0.002** |
| Note: A significant *b*-weight indicates the semi-partial correlation is also significant. *β* represents standardized regression weights. *sr^2^* represents the semi-partial correlation squared. *LL* and *UL* indicate the lower and upper limits of a confidence interval, respectively. ** *p* < .01, *** *p* < .001 | | | | | | | |

**Supplement S8. Analyses for major facets of schizotypy and delusion-proneness**

**S8a**. Regression results (drug use) for major facets of schizotypy (log-transformed)


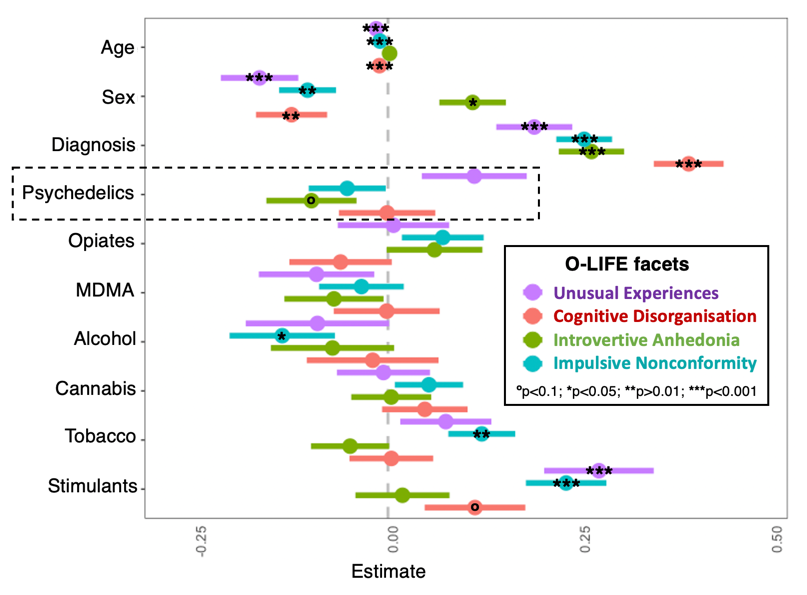


**S8b**. Regression results (drug exposure) for major facets of schizotypy (log-transformed)


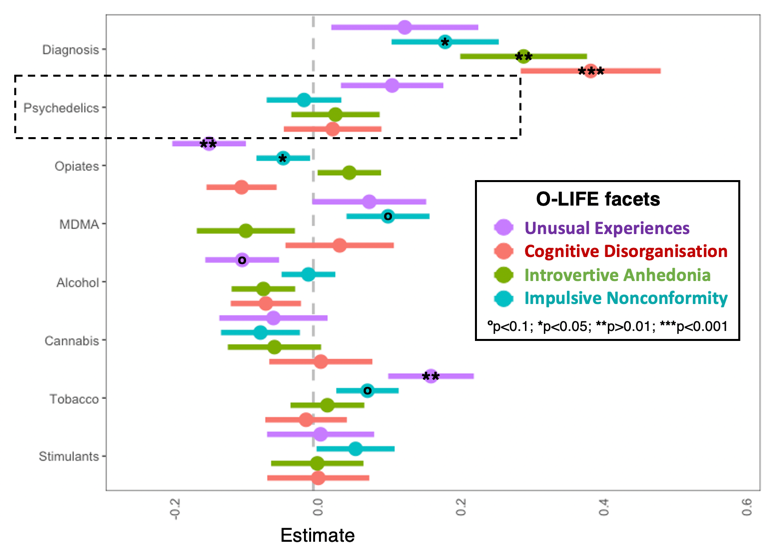


**S8c**. Direct group comparisons for major facets of schizotypy and delusion-proneness (log-transformed)


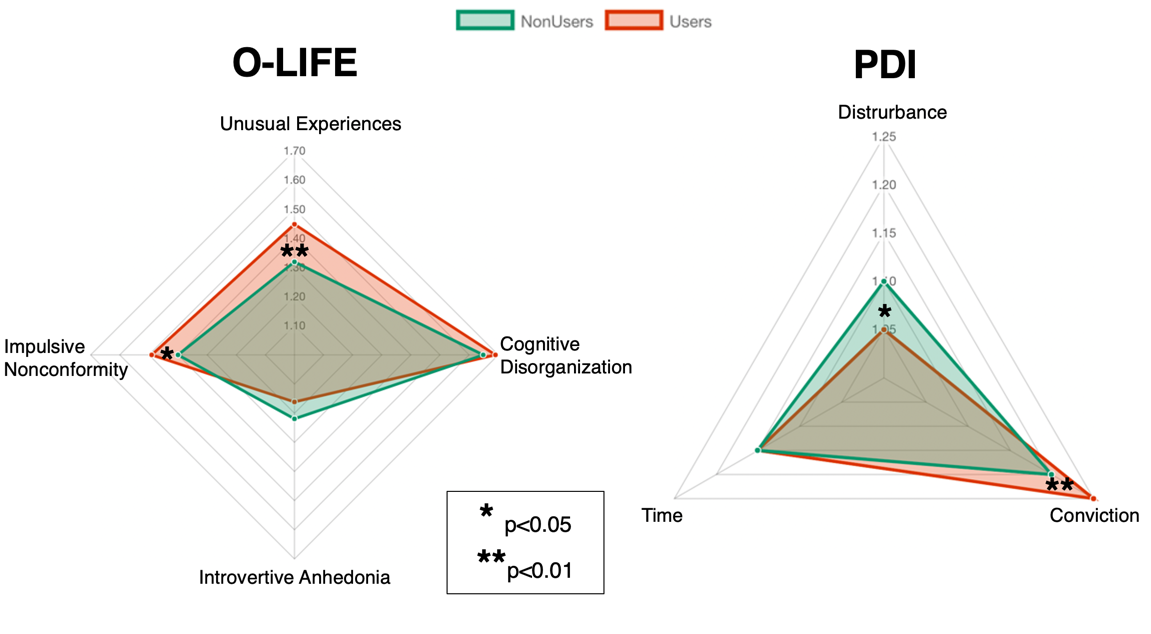


**Supplement S8.** Concomitant drug use in the experimental arm of the study (tried at least once)

|  | **Psychedelis** | **Alcohol** | **Tobacco** | **MDMA** | **Cannabis** | **Stimulants** | **Opiates** |
| --- | --- | --- | --- | --- | --- | --- | --- |
| **PSY-** | 0 (0%) | 12 (71%) | 9 (53%) | 3 (18%) | 6 (35%) | 1 (6%) | 0 (0%) |
| **PSY+** | 22 (100%) | 21 (95%) | 21 (95%) | 12 (55%) | 21 (95%) | 10 (45%) | 4 (18%) |

PSY–: Psychedelic non-users

PSY+: Psychedelic users

**Supplement S9.** Computational model parameters and fit.

|  | **Non-Users** | | **Users** | |
| --- | --- | --- | --- | --- |
|  | **α** | **ρ** | **α** | **ρ** |
| **Pupillometry** | 0.087(0.047)*10^-2^ | 0.82(±0.14) | 0.129(0.043)*10^-2^ | 0.90121(±0.069) |
| **SCR** | 0.71(±0.283) *10^-2^ | 0.50(±0.007) | 0.563(±0.16)*10^-2^ | 0.52(±0.007) |

**α** - learning rate; **ρ** - effect of instructed reversals

**Pupillometry Model**

Response~EV (within-subject effect nested in task version): F_1,3001_= 594.39, p < .0001;

R-squared_marginal/conditional_ = 0.21/0.22;

**SCR Model**

Response~EV (within-subject effect nested in task version): F_1,2922_= 7.30, p = .007;

R-squared_marginal/conditional_ = 0.005/0.22;

**Supplement S10.** Model-derived Expected Value (EV) dynamics


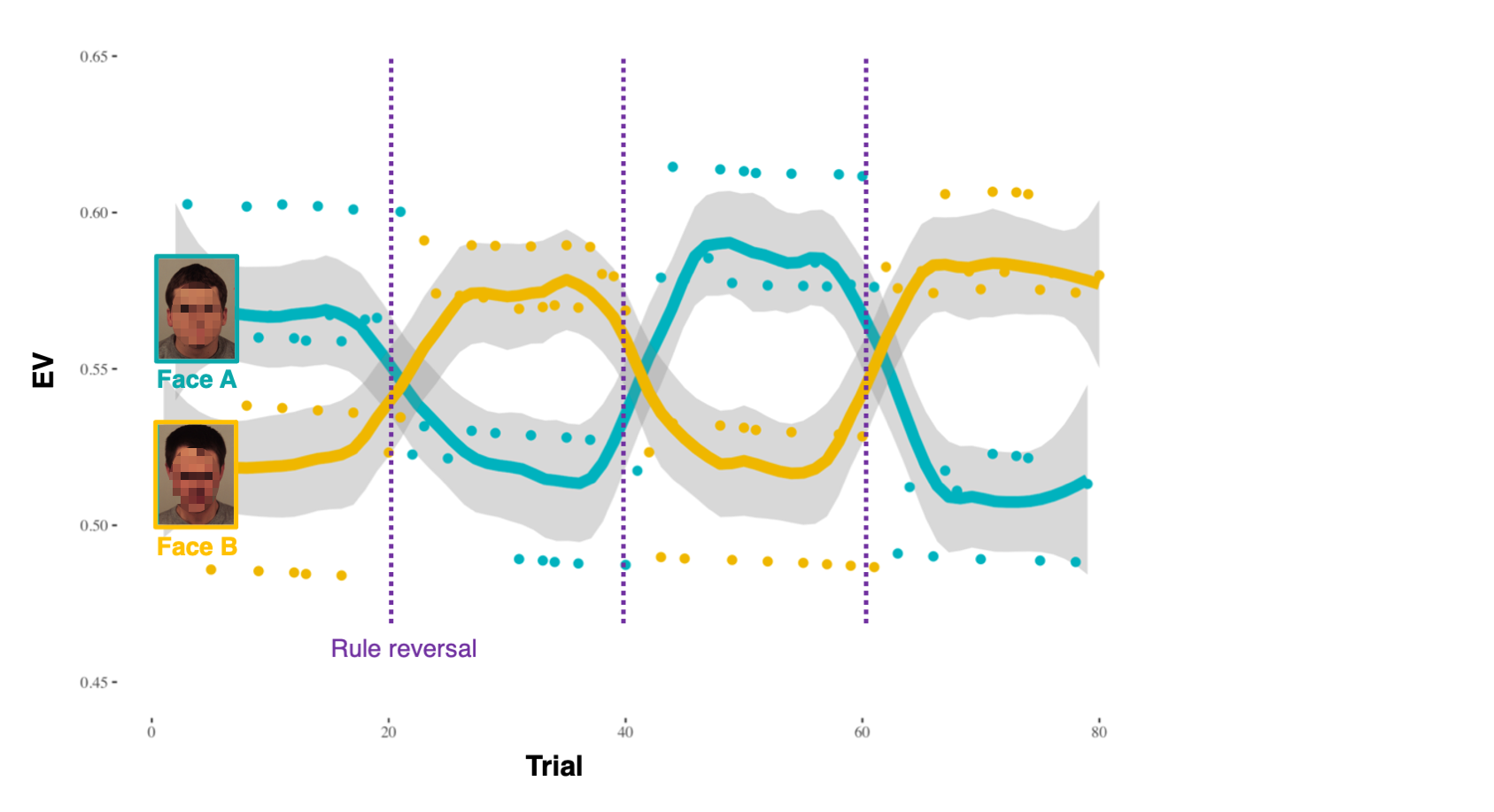


Dynamics of expected value (EV) derived from the model fitted with pupillometry data for one of the two task sequences.

**Supplement S11.** Aversive learning: experimental setting and computational modelling.

**Equipment.** Shocks were delivered through electrodes placed on the right forearm (bar length: 30mm, lead size: 2 x 1.2mm) with salt-free electrode gel for conductance. Delivery of shocks were controlled with DC-pulse electric stimulation devices (MPA100A and MP150; Biopac Systems). The experiment was designed to follow a trace conditioning protocol with a total trial duration of 12 seconds, in which the faces were presented for four seconds and the shocks were delivered in a time-jittered (0.5-2 seconds) manner following the offset of the stimulus. Every shock lasted for 180ms. The experiment was presented with PsychoPy (version 1.90.1).

Before starting the task, subjects placed their chin on a chin rest to ensure no moving of their head during the task, after which their gaze was calibrated. Pupillometry data from both eyes were collected with an eye-tracking device (RED250mobile, SensoMotoric Instruments), with a sampling frequency of 250Hz. Pupillometry data underwent a number of standardized preprocessing steps: 1) removal of extreme signal values (<1mm and >9mm), 2) linear interpolation of the missing data-points, and 3) de-trending. A peak change in a window following stimulus offset was used to quantify trial-by-trial responses ^22^.

Biopac Systems was used to record SCR. Electrodes (27 x 36 mm) were attached to the distal phalange of the left index- and middle finger, electrode gel containing 5% NaCl (salt) was used for conductance. The data were processed and analyzed using Ledalab (version 3.4.9); manual artefact correction was performed when needed, followed by smoothing (adaptive smoothing). Each trial was quantified as the trough-to-peak amplitude within 0.5 to 4.5 seconds after stimulus onset. Responses below 0.01 microSiemens (μS) were regarded as non-specific SCRs.

**Computational Modelling.** A modified Rescorla-Wagner (RW) model that addresses effects of instructed rule reversals was employed to analyze fear learning patterns. The classical RW model describes trial-by-trial dynamics of expected value (EV), which is, in our case, assumed to be proportional to the measured fear responses (pupil diameter and SCR). Electric shocks were used as reinforcements; an increase in EV thus indicates that a shock is predicted to occur with higher probability. The EV is updated when there is a prediction error (PE, i.e. a mismatch between subjects’ expectations and an actual event), and the learning rate (*α*) determines how promptly it is updated. In the classical RW formula below, *V* *=* EV, *r* = 1 denotes a shock, *r* = 0 denotes no shock, *x* denotes cue, *t* denotes trial, and *δ* denotes PE. The PE was calculated as the sum of the EV of the current cue, minus 1 or 0, (shock or no shock):

*V_t+1_(x_t_) = V_t_(x_t_) + αδ_t._  | V_0_ was set as a free parameter estimated from SCR data for each individual*

*δ_t_ = r_t_ – V_t_(x_t_)*

Since the RW model describes learning from reinforcement only, and does not address effects of instructions on fear response reversals, we introduce an additional parameter *ρ*, which determines the extent to which the expected values of the cues are reversed upon receiving a corresponding information:

*V_t+1_(x_a_) = ρ∗V_t_(x_b_) + (1 − ρ)∗V_t_(x_a_)*

*V_t+1_(x_b_) = ρ∗V_t_(x_a_) + (1 − ρ)∗V_t_(x_b_)*

Upon instructions, the EV for cue *x_a_* was calculated as the sum of its value multiplied by 1 – *ρ*, plus the value of *x_b_* multiplied by *ρ*. If *ρ* = 1, cue *x_a_* obtained the value of cue *x_b_*. If *ρ* = 0, each cue kept its original value, and vice versa upon instructions of the EV for cue *x_b_*. In other words, the EV of the CSs switched upon instructions about rule reversals (*ρ* = 1), while they remained the same if contingencies did not reverse (*ρ* = 0), and learning continued through reinforcement only. *ρ* was modelled as a free parameter, and since subjects were instructed about contingencies before initiation of the first trial, initial EVs were modelled as free parameters as well. Model parameters were estimated using Markov Chain Monte Carlo (MCMC) methods.

All analytical steps were implemented in the R programming environment (*R Core Team, 2019*) using *rstan* package for model implementation and MCMC.

**Supplement S11.** Correlation plot between schizotypy composite score and the included questionnaires.


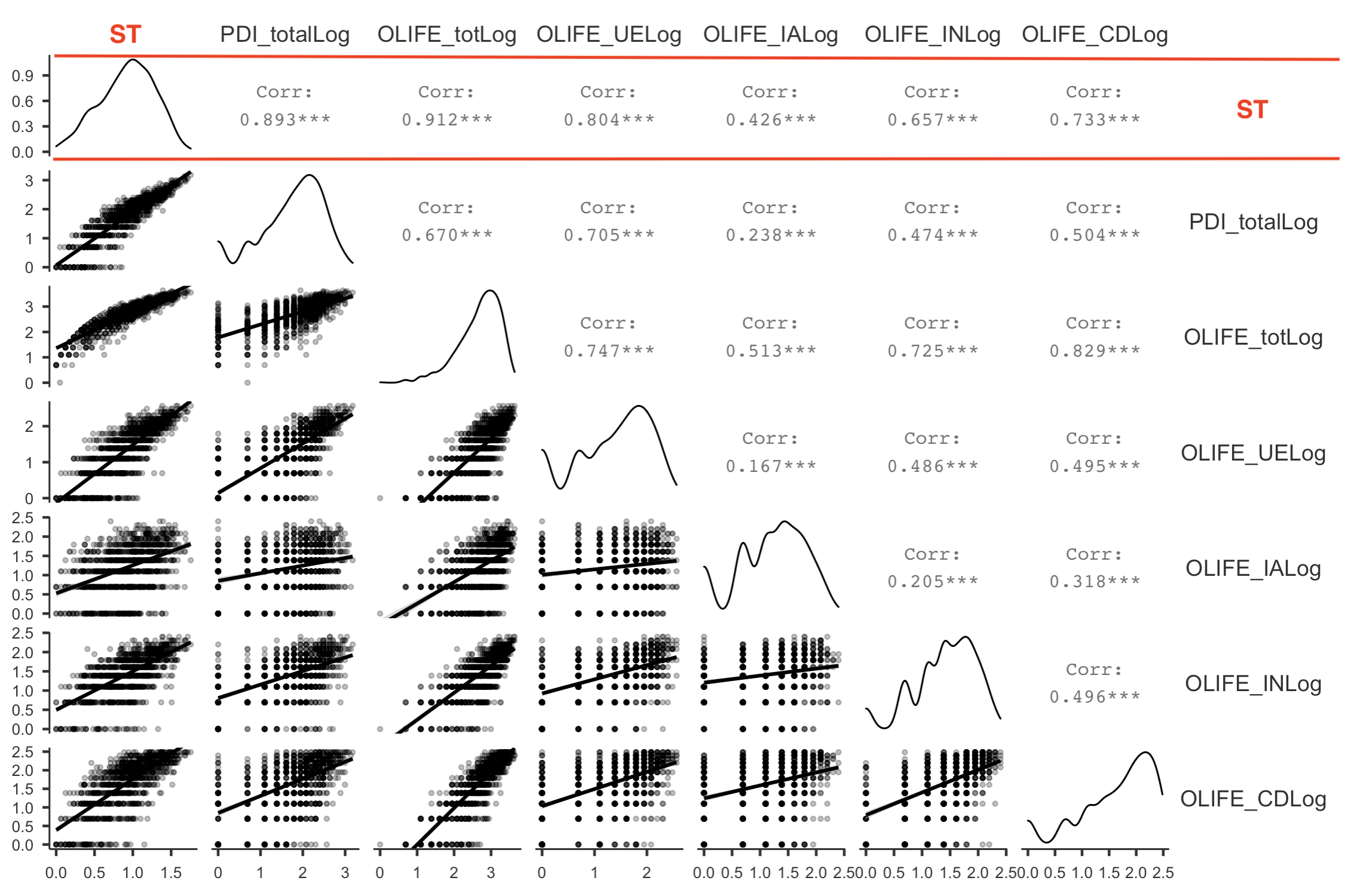


ST – schizotypy composite score merging effects of the PDI and OLIFE total scores; PDI+ – extended Peters Delusion Inventory; OLIFE – 43-item Oxford-Liverpool Inventory of Feelings and Experiences; UE, IA, IN, CD – subdomains of OLIFE (Unusual Experiences, Introvertive Anhedonia, Impulsive Non-conformity, Cognitive Disorganisation, respectively
